# An attachment research perspective on ADHD

**DOI:** 10.1007/s40211-016-0182-1

**Published:** 2016-06-09

**Authors:** Ruediger Kissgen, Sebastian Franke

**Affiliations:** Faculty II Developmental Science and Special Education, University of Siegen, Adolf-Reichwein-Str. 2, 57068 Siegen, Germany

**Keywords:** Attachment theory, Child and adolescent psychiatry, ADHD, Attachment research, Bindungstheorie, Kinder- und Jugendpsychiatrie, ADHS, Bindungsforschung

## Abstract

Since the beginning of clinical attachment research in the mid-1980s the number of research projects in this area has been continuously increasing. The research questions so far can be allocated to numerous medical disciplines such as psychosomatic medicine, adult psychiatry or child and adolescent psychiatry. Recently, children with ADHD and their families have also become subjects of this branch of research. Their specific behavioral characteristics from early childhood on constitute unique challenges on the parent-child interaction. If these interactions develop in a suboptimal way, children may develop an insecure or even a disorganized attachment quality. The latter represents a risk factor for a clinically significant psychopathological development.

This article initially presents basic principles of attachment theory and discusses the relevance of the cardinal symptoms of ADHD for clinical attachment research. Subsequently, it outlines and discusses the main results of existing research regarding attachment and ADHD. It concludes with a perspective on research questions that need to be addressed in the future with regard to a transgenerational model that highlights the importance of parental attachment representations to the development of children’s attachment quality.

## Zusammenfassung

Seit Beginn der klinischen Bindungsforschung in der Mitte der 80er-Jahre hat die Anzahl der dort angesiedelten Forschungsprojekte stetig zugenommen. Die mittlerweile bearbeiteten Fragestellungen können zahlreichen medizinischen Fachrichtungen wie beispielsweise der Psychosomatik, der Erwachsenenpsychiatrie oder auch der Kinder- und Jugendpsychiatrie zugeordnet werden. Auch Kinder mit ADHS und ihre Familien sind inzwischen zum Gegenstand dieses Forschungszweiges geworden. Ihre ab der frühen Kindheit auftretenden Verhaltensbesonderheiten stellen besondere Herausforderungen an die Eltern-Kind-Interaktion. Entwickelt sich diese suboptimal, dann können Kinder eine nicht-sichere oder gar desorganisierte Bindungsqualität entwickeln. Letztere entspricht einem Risikofaktor für eine klinisch bedeutsame psychopathologische Entwicklung.

Der Beitrag klärt zunächst bindungstheoretische Grundlagen und erörtert anschließend die Relevanz der Kardinalsymptome von ADHS für die klinische Bindungsforschung. Nachfolgend werden die Hauptbefunde vorliegender Forschungsarbeiten zum Thema Bindung und ADHS skizziert und bewertet. Den Abschluss bildet ein Ausblick auf Forschungsfragen, die es künftig zu bearbeiten gilt. Letzteres geschieht unter Einbezug eines transgenerationalen Modells, in dem die Bedeutung der elterlichen Bindungsrepräsentation für die Entwicklung der kindlichen Bindungsqualität aufgezeigt wird.

## Attachment theory and attachment research

Attachment theory as developed by Bowlby [1–3] is a model of human development based on psychoanalysis and ethology with a systems theory orientation. Bowlby suggested that a baby is phylogenetically determined to attach itself in the course of the first year of its life to his main caregivers. If a child suffers stress, separation, or danger, its attachment system is activated. In this state of activation every child has a highly effective repertoire of signals to activate the caregivers’ caregiving system. Triggered by the child’s attachment signals, the attachment figure will try to regulate the child. In the course of this routine the child develops an inner working model (IWM) of attachment. As Bretherton [4–7] stated, IWMs can be regarded as generalized representations of events in the sense of ‘lived experiences’. If an attachment figure perceives the children’s attachment behaviors and everyday signals in an appropriate way, if he interprets them correctly and finally acts adequately and promptly, he displays sensitive behavior according to the definition of Ainsworth et al. [8]. The child will picture such an attachment figure in its IWM as competent, reliable, and predictable and it will attach itself securely to this person in the course of the first year of its life. If a child often experiences its signals to be unresponded or misinterpreted, and that the attachment figure reacts inappropriately or too late, it will develop an insecure-avoidant attachment to this person. Though it will picture this attachment figure in its IWM as acting inadequately and as not reliable, the child is able to predict the reactions of the person to its signals just as precisely as a securely attached child does. A child with an insecure-ambivalent attachment fails on this. Such a child will experience this attachment figure as unpredictably alternating between competent, reliable, and predictable and in other situations inadequately and unreliable. Twelve years after Ainsworth et al. [9] had first described this typology of organized attachment patterns it was extended to include the disorganized attachment quality [10, 11]. If a child is neglected, maltreated, or abused, if it experiences its attachment figure to fall mentally ill, if it is threatened or traumatized otherwise by her, it will develop a disorganized attachment. This attachment quality confronts the child with the dilemma that the person meant to provide relief to the child is identical with the one to threat and endanger it. In the subsequent breakdown of the attachment system the child will act in a frightened, overanxious, petulant and/or hypervigilant way in the presence of this specific attachment figure. As attachment research proved in many longitudinal studies [12–14], early childhood attachment experiences are highly predictive with regard to later psychosocial development and level of functioning (self-image, self-esteem, social competence, cognitive ability) up to adulthood. In the meantime, common agreement exists concerning the fact that a secure attachment in the first year of life features as a major protective factor with respect to the psychosocial development of the child while the disorganized attachment poses a risk factor for subsequent psychopathological development [15].

Attachment research initially focused on childhood and the first assessment procedure – the Strange Situation – was developed to evaluate attachment qualities in 11–20 months old infants [16]. Subsequent assessment procedures like the Attachment Q‑Sort [17] or the Attachment Story Completion Task [18] and the Child Attachment Interview [19] expanded the perspective on the preschool and school age. While these instruments merely depict a selection of a wide range of assessment procedures in childhood, the Adult Attachment Interview (AAI) is the procedure that enables close scrutiny of adult attachment representations [20]. The AAI is an autobiographical semistructured clinical interview in which participants are questioned with respect to their childhood attachment relationships. Recently a less time-consuming projective procedure for classifying attachment representations has been introduced, the Adult Attachment Projective (AAP) [21–23]. In the AAP the testee is presented with pictures showing various scenes, with the intention of activating the persons’ attachment system. The analysis of the verbatim protocols is concluded by the classification of the attachment representation. Participants classified as autonomous (in childhood: secure attachment) demonstrate a flexible approach to attachment-relevant feelings. Lack of free emotional approach to attachment-relevant feelings and minimization or deactivation of attachment needs lead to assignment of the dismissing category (in childhood: insecure-avoidant attachment). Participants classified as preoccupied (in childhood: insecure-ambivalent attachment) present hyperactivated attachment needs and extremely emotionalized narratives. The interviews classified as unresolved (in childhood: disorganized attachment) contain narratives of emotional disorientation and linguistic incoherence in attachment-relevant narratives.

Since these inventories were introduced into attachment research, numerous studies were carried out concerning the prevalence of the various attachment representations in normative and in clinical samples. A meta-analytical evaluation of these studies by van IJzendoorn and Bakermans-Kranenburg [24] resulted in 55 % autonomous, 16 % dismissing, 9 % preoccupied und 19 % unresolved attachment representations for nonclinical mothers. The clinical samples showed the following distribution: 8 % autonomous, 26 % dismissing, 25 % preoccupied, and 40 % unresolved. Compared to the standard distribution in nonclinical groups, the combined clinical groups therefore showed an extremely deviating distribution with a strong bias towards insecure and unresolved participants (χ^2^ = 114.83, *p* < 0.001). It has to be noted that these studies encompassed a great diversity of samples [18]. The authors, however, concluded that it was irrelevant for the overall distribution whether the clinical pathology occurred in the examined adults or in the children [25].

Besides the question of the distribution of attachment representation in adults, attachment research dealt with the transgenerational transmission of parental attachment experiences. The correlations between parental attachment representation and the attachment quality of the child, meanwhile established in numerous studies, are regarded as evidence for the intergenerational transmission of attachment [25–33]. Summing up parental attachment representation and maternal sensitivity as demonstrated in the interaction with the child (see above) are regarded as agents promoting intergenerational transmission.

## ADHD

Since its introduction as a diagnosis, attention deficit hyperactivity disorder (ADHD) has increasingly become a term used to describe a wide range of behavior problems in children. There is considerable disagreement both with regard to the etiology as well as suitable therapy for ADHD [34]. The ICD-10 [35] differentiates between simple disturbance of activity and attention (F90.0) and hyperkinetic conduct disorders (F90.1), as well as other or unspecified hyperkinetic disorders (F90.8 and F90.9). This division is based on the main characteristics of inattention, hyperactivity and impulsivity and assigns a separate category to children that display a particularly conspicuous interaction with their social environment in an attempt to take the heterogeneous aspects of this disorder into account. The further development of DSM-5 [36] included only a few new features of this disorder compared to DSM-IV TR [37]. While the symptom categories inattention and hyperactivity/impulsivity remain, the various subtypes are conceptualized as presentations whose expressions may change during the lifespan. Moreover, ADHD can be diagnosed in adulthood and is placed in the category of neurodevelopmental disorders to reflect correlates between brain development and ADHD.

Overall, this clearly demonstrates that a plethora of different symptom types is combined with each other in the entire picture of ADHD, and that the expressions of this disorder must be considered in a differentiated manner. Although the new features in DSM-5 consider a multifactorial genesis of ADHD, they still do not allow for any new assumptions about a specific etiology. Over the last decades, research has increased which, alongside neuropsychological and cognitive deficits, could also identify factors in the family context of the affected child [38, 39]. In the course of this work, some researchers have theoretically dealt with the connection between attachment and ADHD [40–43] and found parallels between the core symptoms of ADHD and findings from attachment research. Below we will outline main results of the studies that tried to merge these research lines as well as recommendations for future research.

## Attachment research relating to ADHD

It can be assumed that from their first years, children with ADHD present a major challenge to everyday family life. Their inattention, their hyperactivity and their impulsivity impede the primary caregivers in their approach to these children’s signals. Since the caregivers sensitivity in dealing with the childrens signals represents the basis of forming a secure attachment, the requirements for the development of a secure attachment appear to be less favorable than in children in unaffected control groups.

Moreover, it could be demonstrated that insecurely attached individuals are more susceptible to problems with emotional regulation and behavior regulation [44]. Problems of self-regulation (e. g. impulse control, inhibition, settling down) are likewise central elements of the ADHD syndrome, which is sometimes even conceptualized as disorder of self-regulation [45–47]. From this, it can be deduced that early interactions between primary caregiver and child influence the impairment of self-regulation in children with ADHD [46, 48].

In addition, literature suggests that attachment security has a positive effect on certain areas of competency children with ADHD have difficulties with [49]. Secure attachment is associated with enhanced performance in attention-related tasks and an increased attention span [50, 51]. Matas, Arend and Sroufe [52] found greater enthusiasm, extended perseverance, more willingness to cooperate as well as higher effectiveness in securely attached children than in those insecurely attached. Likewise, attachment security in early childhood is associated with cognitive impulse control, task orientation and delay of gratification at the age of six years [53, 54].

In contrast, disorganized attachment in early childhood shows a strong connection with a later abnormality or psychopathology [14, 55]. Although research on the association between attachment and ADHD is very limited, the few studies in this area nonetheless predominantly find a connection between disorganized attachment and ADHD [56–59].

In a sample of 100 8½ year old children, Thorell et al. [59] demonstrated a relation between disorganized attachment and ADHD symptoms. These symptoms were assessed one year after rating attachment representations. The association persisted irrespective of externalizing behavior problems, measured using the Children’s Behavior Questionnaire [60], as well as executive functioning. The independence of the cognitive functions measured suggest that the ADHD symptoms themselves are associated with disorganized attachment rather than cognitive deficits, which can occur in the course of ADHD. With regard to the behavior problems, the authors point out that although there is a strong overlap between externalizing behavior problems and ADHD [37, 61], factor analyses nevertheless confirm a conceptual difference between the two constructs [62]. Thus, in the investigation of the connection between attachment and ADHD, controlling for externalizing behavior problems is essential. The study by Thorell et al. [59] was explicitly limited to the investigation of disorganized attachment; due to the low correlation between attachment insecurity and ADHD symptoms (r = 0.14, n.s), the authors exclude a closer investigation of this association from their analyses. They conclude that the lack of independence of the influence of attachment insecurity on ADHD precludes a connection between these two constructs. In other publications, however, definite indications of such a connection can be found [49, 63].

The latest study on this topic by Scholtens et al. [58], using a sample (*n* = 89) of 6–10 year olds and a short narrative story stem technique, likewise found significantly stronger expressions of ADHD in disorganized children than in children who were securely attached. This connection could not be accounted for either by the overlap between ADHD symptoms with externalizing behavior problems or by cognitive deficits. These latest results may suggest a specific connection especially between attachment disorganization and ADHD. However, the particular quality of this tie is not understood or even characterized yet.

## Conclusion

Even though individual research projects were able to underpin some theoretical considerations empirically in recent years – mostly within the connection of disorganized attachment and ADHD – results are very heterogeneous. Hence, the link between attachment and ADHD is still not demonstrated. One weakness of previous research on this topic lies in the general consideration of the construct ADHD. The various phenotypic expressions of this disorder are not taken into account, but are rather conceived as a global clinical construct. The resultant heterogeneity of the specific samples allows only poorly differentiated and inconsistent assertions about the relationship to other constructs. Instead, individual aspects of the disorder should first be considered separately in order to link specific symptom classes with respective constructs.

In the above mentioned paper, Erdman [40] suggests a framework that views children’s behaviors as a contextual response to parental attachment, stressing the importance of the function that a behavior has in a certain context. That perspective does not doubt the existence of ADHD, but it assumes that it is frequently misdiagnosed and that a child might as well display ADHD-like behaviors in order to maintain a specific parent-child relationship with the aim to keep it organized. Erdman argues that these behaviors might then be interpreted as being a sign of ADHD. This argument for one supports the differentiated examination of ADHD and its specific behaviors. Furthermore, it highlights the role of the context, specifically the parents’ behaviors in developing certain constellations of interaction that can be viewed as detrimental for the child.

One important variable that might affect the specific parent-child relationship is the parents’ own attachment representation. As discussed above, these representations play a major role in the intergenerational transmission of attachment. The contextual model of van IJzendoorn and Bakermans-Kranenburg [25] may identify the aspects contributing to the attachment-relevant experiences of a child. In the model, (1) early childhood experiences of parents with their own parents within the family context precede (2) further attachment experiences during childhood in other contexts. Based on these experiences IWMs of attachment are constructed which during adolescence lead to a dominant IWM of attachment, figuring as (3) attachment representation. The adult attachment representation and (4) its social context influence (5) parenting behavior. Ultimately the parenting behavior and the (6) specific child characteristics will implement the child’s future attachment experiences. A first study fitting parts of this model focused on attachment representations in mothers of children with ADHD. Kissgen et al. [64] showed that the prevalence of maternal insecure and unresolved attachment representations increases with the degree of severity of children’s ADHD symptoms.

According to these preliminary considerations, from an attachment perspective, there are two major aspects research should focus on. One should address the role of transgenerational transmission of attachment in the development of ADHD in the child. The second one should investigate whether attachment representations of children with ADHD differ from those of children without ADHD. If that is the case, the specific behaviors within the construct of ADHD that contribute to that difference need to be identified, since some behaviors might be the adequate contextual response to an impaired relationship with the parent. In doing so, it is important to keep possible mediating and moderating factors in mind that might also account for the relation between these two constructs. All these considerations are of great importance in the investigation of ADHD and attachment and can help to provide a more detailed and differentiated perspective on the extent of their mutual influence.
